# Healer’s Art in the Online Era: Successes, Challenges, and Implications

**DOI:** 10.1007/s40670-024-02272-w

**Published:** 2025-01-13

**Authors:** Jeannette K. Manger, Alyssa C. McManamon, Amber Todd, Adrienne Stolfi, Dean X. Parmelee, Evangeline Andarsio

**Affiliations:** 1 Department of Medical Education, Wright State University Boonshoft School of Medicine, Dayton, OH USA; 2 Department of Internal Medicine, Wright State University Boonshoft School of Medicine, Dayton, OH USA; 3 Department of Medicine, Uniformed Services University of the Health Sciences, Bethesda, MD USA; 4 Department of Pediatrics, Wright State University Boonshoft School of Medicine, Dayton, OH USA; 5 Medical Education, Psychiatry & Pediatrics, Wright State University Boonshoft School of Medicine, Dayton, OH USA; 6 Medical Education and Obstetrics & Gynecology, Wright State University Boonshoft School of Medicine, Dayton, OH USA

**Keywords:** Healer’s Art, Medical education, Online learning, Affective learning

## Abstract

**Purpose:**

Healer’s Art (HART), a health professional elective course, shifted to online platforms during the pandemic year (2020–2021). Because HART focuses on affective domain aspects of such education, the question arose of its validity and efficacy in the online format. This study aimed to identify challenges and experiences of online versus in-person HART learners.

**Methods:**

The authors compared students’ end-of-course evaluations between in-person and online cohorts across 3 years. The evaluations included Likert scale responses compared between cohorts with Fisher’s exact tests. Novel questions with narrative responses in the online cohort’s evaluation captured information on challenges with the online platform. Narrative responses were analyzed using constant comparative analysis.

**Results:**

No difference was found between in-person (*n* = 654) and online cohorts (*n* = 570) in ratings of good/excellent for the overall course (1111/1203, 92.4%), course faculty (1184/1214, 97.5%), and small group experience (1142/1208, 94.5%). Thematic analysis of narrative responses indicated that online HART engagement supported student development of *community, professional identity formation, self-care, and relationships*.

**Conclusions:**

The findings suggest that HART effectively supports affective domain learning in medical and health professional students whether delivered in-person or online. The authors share challenges and successes of online HART, thus increasing the delivery versatility of this course.

**Supplementary information:**

The online version contains supplementary material available at 10.1007/s40670-024-02272-w.

## Introduction

As medical and other health professional schools shifted to online course delivery during the COVID-19 pandemic, students not only struggled with online delivery of material, but also with loneliness and isolation [1–5]. The Remen Institute for the Study of Health and Illness (RISHI) recognized early in the pandemic that the sense of isolation and loneliness experienced by health professions students would magnify, and thus transitioned the Healer’s Art (HART) course to a virtual experience [6]. Although the Healer’s Art course is known to support community and develop the affective domain in students, little is known about the effectiveness of online delivery of such a course [7]. In this study, we examine the successes and challenges of online HART instruction so that institutions can confidently deliver the HART curriculum in a format best-suited to learners’ needs.

HART, designed and pioneered in 1991 by Rachel Naomi Remen, MD, at the University of California San Francisco, is one of the best-known educational strategies to support the development of professional identity formation (PIF) and building of supportive communities among students and faculty [8–12]. It is offered nationally and internationally in over 70 health professions schools and uses a discovery model of learning to address the affective domain with a non-cognitive or “soft skills,” highly interactive, in-person learning approach [13]. HART includes five 3-h sessions that incorporate storytelling, poetry, journaling, creative arts, and reflection within a safe interactional community of students and faculty. Students and faculty rediscover their professional values in medicine and other health professions as they explore ways to nurture their wholeness, honor grief and loss, allow awe and mystery, and embrace service as a way of life [8]. Traditionally, this course has been offered in-person and features small and large group discussions with an emphasis on active student participation. The shift to a virtual experience was done with minimal disruption using online meeting platforms to facilitate large group discussion and “breakout rooms” for smaller group interactions. The timing and course schedule were unchanged. To date, we have no data that compare teaching the HART elective online versus the conventional in-person approach.

The “soft skills” of medicine are becoming more widely addressed in undergraduate medical education, thanks to earlier efforts that encourage the exploration of core values and beliefs. Professional identity formation, an answer to this call, is the iterative process of defining what it means to be a medical professional: the thoughts, values, behaviors, and feelings involved [14]. Researchers have called for explicit attention to professional identity formation through educational objectives rather than the implicit hope that students develop these skills with time and experience [15, 16]. While many of the current approaches to addressing professional identity formation within the medical curriculum focus on reflective writing and narrative reflections, HART incorporates a stronger emphasis on social construction of professional identity through creative arts and storytelling [17].

The Healer’s Art curriculum engages in the affective domain, the non-cognitive space, and offers an educational learning model which explores, develops, deepens, and validates the personal experience of meaning, purpose, calling, service, and community in the health professions. The affective domain educational strategy offers transformative learning for students in the development of their professional identity as it relates to centering values, emotions, and intuition to inform the practice of caring for others. Through experiential reflective exercises and creation of safe interactional space, HART students come to self-discovery of what matters to them and how they desire their values expressed in interactions with their patients and colleagues, thus supporting students in professional identity formation [8]. In fact, participants of the HART course experienced perceived increases in empathy toward patients and peers, commitment to the profession, a more developed idea of what a doctor is, and decreased levels of burnout—and many of these benefits persisted over time [8, 18]. Further, HART provides students with self-care tools and strategies to reaffirm their commitment to the profession, which has been noted as an important aspect of professional identity formation [19]. Should the virtual HART provide effective and sustainable professional identity formation, it opens the door to more flexible course offerings.

With the advent of online meeting platforms and distance learning, many lecture-based courses have been adapted for the online classroom with ease, but one-size-fits-all online learning can be isolating to students [20]. Engagement in online classrooms varies across disciplines, with sciences generating the least amount of student interactions [21]. Active learning poses additional challenges; however, instructors have adapted to the change and fostered student engagement online [22, 23]. Less studied are courses designed to support professional identity and empathy in an online setting. In-person longitudinal empathy education is more effective at shifting student perspectives than a one-time class, and synchronous online classes tend to be more effective than asynchronous classes at developing affective domains in students [24, 25]. Engaging learners in meaningful—and lasting—online interactions is both difficult and necessary. Because community is a critical aspect of engaged online learning, it should follow that a course designed at the unique intersection between humanity and medicine would elicit more authentic student engagement [26]. Other professional identity coursework conducted during the pandemic was met with positive feedback in terms of feeling connected [27], but no study has investigated the comparability of affective learning outcomes in both virtual and in-person settings.

The question of whether this highly successful elective course translates effectively as an online course is important for confidence in HART course delivery [7, 8, 18]. If comparable, this knowledge could broaden the range of schools offering online delivery of similar courses and would enhance the accessibility to students in schools with regional campuses, avoid session cancellations due to inclement weather, decrease transportation costs, and omit travel time for students in rural and metropolitan areas. The objective of this study was to determine whether an online HART course is equal in effectiveness to an in-person HART course, by comparing end-of-course student evaluations. We outline three specific research questions: (1) What are the experiences of students in an online HART course? (2) What are the challenges for students specific to online HART delivery? and (3) How do students’ learnings compare with the in-person course?

## Materials and Methods

### Study Design and Participants

Anonymous student end-of-course evaluations of in-person HART courses conducted in academic years 2018–2019 and 2019–2020 and of online courses (2020–2021) were analyzed using a mixed methods approach. All participating institutions received exempt status from their institutional review boards.

Evaluations included three statements about course quality, nine statements related to the educational experience, and three demographic questions (gender, age, and year in school). Quality statements were measured on 5-point Likert scales from poor to excellent, and experience statements on 5-point Likert scales from strongly disagree to strongly agree. Four open-ended questions were included on both the evaluations. An additional four open-ended questions specific to the online experience were asked of participants of the online course. Table 1 includes all Likert scale responses from both in-person and online student evaluations to items listed under “As a result of this course….” Demographic variables are reported in the results. For all open-ended questions, only the responses from the online course evaluations were included as our research questions focused on the experience of online HART.

**Table 1:** Comparisons of responses of agree or strongly agree to evaluation items under “As a result of this course…”

As a result of this course	Related themes	In-person students *n* (%)	Online students *n* (%)	*p* value
I feel more committed to medicine	PIF	515 (79.6)	433 (83.6)	0.096
I feel more committed to patient centered care	Relationships, Self-care	584 (90.0)	478 (90.2)	0.922
I am more confident that I can be a good doctor	PIF	542 (83.6)	436 (85.8)	0.325
I understand better what being a good doctor entails	PIF	569 (87.7)	466 (89.1)	0.466
I am more certain that I belong to this profession	PIF, Relationships	548 (84.6)	458 (87.9)	0.107
I am clearer about what I personally can offer my patients	PIF, Relationships	556 (85.7)	462 (89.2)	0.078
I feel more supportive of my classmates	Relationships	619 (95.4)	514 (97.9)	0.024

Sample sizes for individual statements ranged from 647 to 649 for the in-person group and 508 to 525 for the online group

### Quantitative Data Analyses

We summarized demographics and Likert scale responses with frequency and percent of non-missing responses. We grouped responses due to small sample sizes for some of the options. We recoded Likert scale responses from poor to excellent into poor-average (numeric options 1–3) and good–excellent (options 4–5). For questions with options of strongly disagree to strongly agree, we recoded responses into strongly disagree-neutral (options 1–3) and agree-strongly agree (options 4–5). For demographics and Likert scale variables, we dichotomized responses and compared between the in-person and online course students with Fisher’s exact tests. We considered *p* values less than 0.05 to be statistically significant. We conducted quantitative analyses with IBM SPSS Statistics for Windows, Version 29.0 (IBM Corp, Armonk, NY).

### Qualitative Data Analyses

We employed constant comparative analyses to identify emergent themes within the open-ended responses. Six independent coders (all authors) reviewed the open-ended responses to identify open codes for each question. Some student responses contained multiple codes. The group conducted axial coding to solidify the themes. Coders then completed selective coding with an interrater reliability of greater than 85%. Disagreements were discussed until consensus was reached. We share the emergent themes in the “Results” section. We conducted qualitative analyses with NVivo 13 for Windows (Lumivero, Denver, CO).

## Results

Thirty-one schools participated in at least one HART course during academic years 2018–2021 and submitted student evaluations for inclusion in the study. There were 28 medical schools (26 allopathic, 2 osteopathic), 1 physical therapy doctoral program, and 2 schools of veterinary medicine; a complete list of the schools is available in Supplementary File 1. Six hundred fifty-four student evaluations were submitted for the in-person courses conducted in 2018–2020, and 570 were submitted for the online course conducted in the 2020–2021 academic year. Sample sizes varied by question because not all students answered all questions. Overall, a higher proportion of students were female (696/1134, 61.4%) and ≤ 24 years old (636/1131, 56.2%), with no differences between the in-person and online courses. First-year medical students made up a significantly higher proportion of the online class compared to the in-person class (329/436, 75.5% vs. 398/631, 63.1%, *p* < 0.001).

### Quantitative Evaluation Data

#### Quality of the Course Faculty, Small Group Experience, and Overall Curse

For the course faculty, 97.5% (1184/1214) of students overall rated them as good or excellent (1053/1214, 86.7% excellent), with no difference between the in-person and online courses (*p* = 0.463). The small group experience was rated good or excellent by 94.5% (1142/1208) of students (930/1208, 77.0% excellent), again with no difference between the in-person and online courses (*p* = 0.164). Ratings of the overall course were also similar between in-person and online, with 92.4% (1111/1203) of students rating the courses as good or excellent (819/1203, 68.1% excellent) (*p* = 0.231).

#### HART Educational and Small Group Experiences

Two evaluation items were statements about the educational and small group experiences, with response options on a 5-point Likert scale from strongly disagree to strongly agree. For the first statement, “Healer’s Art provides educational experiences not available elsewhere in my medical school curriculum,” 94.6% (1139/1204) of students agreed (233/1204, 19.4%) or strongly agreed (906/1204, 75.2%). For the second statement, “Healer’s Art small group experiences are similar to other medical school small group experiences,” most students had responses of strongly disagree-neutral (980/1151, 85.1%). There were no differences between the in-person and online students for either statement (*p* = 1.00 for the first statement, *p* = 0.113 for the second statement).

#### Responses to Evaluation Items Under “As a Result of This Course…”

Table 1 shows the number and percent of in-person and online students responding agree or strongly agree to statements related to the themes of professional identity formation, relationships, and self-care. Most students reported that the course resulted in them feeling more committed to medicine and patient centered care, and more confident or certain about being a doctor (in the case of medical students). There were no differences between the in-person and online students for all but one statement. For “I feel more supportive of my classmates,” a significantly higher proportion of online students (514/525, 97.9%) agreed or strongly agreed with the statement compared to in-person students (619/649, 95.4%).

### Qualitative Evaluation Data

#### Overall Online HART Educational and Small Group Experiences

When analyzing the qualitative data and coding for emergent themes, we noticed that the themes of professional identity formation, community, relationships, and self-care tools were predominant in the student responses. These align with literature demonstrating the same themes for in-person HART experiences [10, 28]. Since one of our research questions focuses on how online experiences compare with the in-person course, we were encouraged to see these same themes when analyzing our data related to the online HART experience. Our codebook with our themes and illustrative quotes containing student number (e.g., SXX) is shown in Table 2.

**Table 2:** Codebook

Emergent codes	Description	Example quote(s)
Professional identity formation (PIF)	Confirmation of values held by the profession, open heart, listening as a physician skill	“I have promised to take the time to listen to my patients and their narratives.” (S203); “I will listen attentively with my brain & heart to others’ stories.” (S295)
- Listening/be present/slow down	Mentions the need to listen or be present or slow down to listen	“Always listen to the patient and the story they have” (S66)
- Humanity	Mentions the need for humanity or seeing people as individuals	“I will never forget each patient is an individual with their own unique story who is coming to me at one of their most vulnerable times” (S51)
- Compassion	Mentions compassion	“I make a promise to always lead with compassion” (S65)
- Vulnerability/openness	Mentions need to be vulnerable or open	“I’ll be comfortable enough with others to share with them” (S82)
- Service	Mentions sense of service	“I won’t turn a blind eye to those in need” (S350)
- Respect	Mentions respect	“I can treat them with the respect they deserve” (S57)
- Be non-judgmental	Mentions need to be non-judgmental or withhold judgement	“… and to the best of my ability not make any judgments before I can listen” (S333)
- Be authentic	Mentions authenticity	“… to be authentic in my knowledge, doubt, sadness, joy, and all emotions in between” (S338)
- Humility/humbleness	Mentions humility or humbleness	“Allow me to retain humility and awe every day” (S337)
Relationships	Relationships between physician–patient, physician-physician, and/or physician-healthcare team	“I want my patients to feel comfortable around me and not feel like I am only there to fix their anatomical issue.” (S123)
Community	Vulnerability with peers, safe space, sharing my story and listening to peers’ stories	“Understanding that many of my peers are facing the same thought and challenges is reassuring.” (S345); “I was able to open up with peers about some of my struggles and concerns.” (S029)
Self-care tools	Self-care tools to nurture and strengthen resiliency; self-nurturing	“The journaling tools I left with.” (S025)
How HART not valuable: loss of connection due to online	Negative toward the online course because of loss of connection doing it online	“It feels harder to connect with someone over a monitor.” (S044)
How HART not valuable: online format (not specific)	Negative toward the online format of the course in general	“I think it was harder with the virtual format” (S152)
How HART not valuable: time too long	Mentions that the time for course or sessions was too long	“It was a decent segment of time to sacrifice from study time.” (S186)
How HART not valuable: large group experience	Negative toward large group experience	“I didn’t find the large group exceptionally valuable.” (S472)
How HART not valuable: disorganized	Mentions that course/sessions were disorganized	“I wish that there was a little more structure and questions etc. At times it felt awkward or I didn’t know what to share because the questions were so broad or it just felt like that was not that much to share.” (S115)
How HART not valuable: time too short/time in general	Mentions that the time was either too short or mentions time in general with no specifics	“It felt cut short, which is natural, but was very quickly personable and then ended.” (S542); “Time.” (S539)
How HART not valuable: not relevant	Course/sessions were not relevant	“I was expecting to learn/be experiencing more content directly about medicine, but it was more just general life things which I can get from my therapist outside of counseling.” (S321)
How HART not valuable: too early in training	Mentions that the course would have been more helpful in later years of training	“… I would likely have benefitted from it more during clinical rotation years of medical school.” (S167)
How HART not valuable: “too heavy”	Mentions that sessions or topics were “heavy”	“Sometimes the topics were heavy, and I personally would have liked a forewarning before the sessions that we were asked to share/think about our grief stories.” (S434)
How HART not valuable: too personal	Negative toward sharing personal information	“I also would have rather not shared deep things with one of my group members.” (S125)
How HART not valuable: too much silence	Mentions excessive silence	“It seemed like a waste of time to sit in so much silence.” (S192)
How HART not valuable: small group experience	Negative toward small group experience	“I think sometimes the small group felt unguided.” (S115)
How HART not valuable: too much religion	Mentions religion in a negative way	“I did not like the ‘awe’ sessions, as there were some vaguely religious undertones which made me uncomfortable.” (S314)
How HART was not valuable: too focused on grief	Mentions excessive focus on grief	“I thought two full sessions on grief was redundant. We didn’t have much to discuss for the second session.” (S194)
How HART was not valuable: everything was valuable	Mentions that everything was valuable or that nothing was not valuable	“Even the parts of Healer’s Art that placed me outside of my comfort zone were still very valuable for me! I can’t think of anything that was not helpful.” (S476)
Challenges with online format: emotional connection	Mentions difficulties in emotional connection due to online format	“I do think it was much harder to connect with my group members as fellow humans rather than just people to whom I owed a story.” (S231)
Challenges with online format: engagement	Mentions difficulties engaging in session due to online	“Sometimes it was hard to stay focused just with life happening around me in my house.” (S368)
Challenges with online format: technical	Mentions technical challenges	“Some group members had issues with their connection” (S254)
Challenges with online format: confidentiality concerns	Mentions confidentiality concerns	“My living situation makes privacy very difficult as the walls are so thin, the house is small, and at any given time I have up to 5 other family members possibly within earshot of what I say, even from my own bedroom. I felt I had to hold back from saying everything I wanted because of this.” (S252)
Challenges with online format: no difficulties	No difficulties with online format	“I thought the virtual format worked well” (S221); “No” (258)

Students completing HART in-person or online are queried on the most valuable thing they learned from the course: “about yourself, about your classmates, about medicine?” For the online HART cohort, students were additionally asked in what ways the online HART learning experience was most valuable and was not valuable.

Within 465 student responses, 510 coded references reported on the most valuable thing they learned from participation in online HART (Q6, Table 3). Many (211, 41.4%) of the references related to PIF. A quarter of the references related to a sense of community; self-care tools and relationships were also themes.

**Table 3:** Overall learnings: most valuable thing learned and how online HART was valuable

Theme	Most valuable thing learned (Q6; 465 student responses)	How online HART was valuable (Q29; 444 student responses)
	*n* (%)	*n* (%)
Community	132 (25.9)	220 (57.0)
Professional identity formation (PIF)	211 (41.4)	114 (29.5)
Relationships	71 (13.9)	0 (0.0)
Self-care tools	96 (18.8)	52 (13.5)
Total coded references	510	386

Within 444 student responses, 386 coded references reported on how online HART was most valuable (Q29, Table 3). Students largely referenced community and professional identity formation, with fewer references to self-care tools and none to relationships.

Within 389 student responses, 435 coded references were about promises or commitments of how they will care for future patients (Q8). The overwhelming majority of references related to professional identity formation (374/435, 86.0%); self-care, relationships, and community were less referenced. Under the theme of professional identity formation, subthemes were generated and included listening/be present/slow down (115/374, 30.7%), humanity (82/374, 21.9%), and compassion (76/374, 20.3%); vulnerability/openness, service, respect, be non-judgmental, be authentic, and humility were less referenced.

Within 409 student responses, 334 coded references shared how their online HART small group experience was different from other (e.g., medical) school small group experiences (Q4). The overwhelming majority of references related to community (298/334, 89.2%); other references included professional identity formation (27/334, 8.1%), self-care tools (5/334, 1.5%), and relationships (4/334, 1.2%).

We were pleased to find the themes of professional identity formation, community, self-care, and relationships within our data. This illustrates that the online HART provides a comparable experience to the in-person HART, and answered our third and final research question. Students shared:

With COVID, I think I have lost a sense of being able to connect with other people. I remembered how valuable this is during the Healer’s Art course. (S39, community).

The most valuable thing I learned is that I am not alone. The school is full of others who are going through the same struggles that I am, and I can rely on anyone anytime. (S50, community).

I have learned how to approach deeper conversations with my classmates and how to develop stronger relationships with my friends, but also how to apply this to patient care and being empathetic with a patient and meeting them where they’re at. (S119, community).

I learned so much about the beauty and power of vulnerability, and the gifts and insights that are offered when we can be vulnerable together – it is a powerful reminder that we are all human, we all have our own stories and although we walk our journeys alone, we can walk beside one another offering compassion, support, caring and love. (S362, community).

Medicine is truly a healer’s art and I will never forget that. It is not just technical and practical but it is full of soul and gratitude. (S11, professional identity formation).

Don’t be afraid to go the extra mile and empathize with your patients. Cherish your experiences and the stories you hear. (S28, professional identity formation).

That empathy and emotions are no obstacles to being a good physician; rather, they are a gift that will only enhance my ability to care for my patients. (S31, professional identity formation).

I have promised myself that I will allow my patients space to express themselves even if that means sitting in an uncomfortable silence for a moment to allow them to feel comfortable sharing. (S75, professional identity formation).

#### Online HART Educational Experiences

After reviewing the questions related to HART educational and small group experiences, we then reviewed the questions focused on the online component, getting at our research questions about the challenges of an online HART course and how their experiences compare with the in-person course.

Interestingly, of the 462 student responses to “Why did you sign up for this course?” only 5.4% (25/462) referenced they had done so wholly or in part due to the pandemic or related online learning concerns (Q11). Exemplary responses included, “Because I wanted to better connect to others in this pandemic…,” “I wanted to feel the community that [my school] has to offer. I find with online learning it’s hard to see the community, but now I feel the community,” and “I wanted an opportunity to remind myself why I decided to pursue medicine: people. In the first year of medical school, especially during COVID-19, it can be very disheartening and discouraging to sit in front of a computer screen for several hours each day.”

Overall, fewer students responded to the question about how online HART was not valuable (*n* = 293) than to other questions. There were 263 coded references for ways online HART was not valuable for students (Q30, Table 4). Thirty student responses did not fall into any of the emergent subthemes. Fewer than half were specific to the online format, slightly more were focused on HART in general, and a few reported that everything was valuable. Of the references that were specific to the online format, nearly 60% cited loss of (human) connection due to online and just over 40% mentioned the online format but did not further elaborate. References that focused on HART in general included a most-shared (35%) concern of time being too long with other responses coded 10% or less.

**Table 4:** Challenges with HART online format and in general

Theme	How online HART not valuable (Q30; 293 student responses)		Challenges with online format (Q31; 435 student responses)	
	*n* (%)	Subtheme *n* (%)	*n* (%)	Subtheme *n* (%)
Specific to online format	115 (43.7)		228 (55.1)	
- Loss of connection due to online		68 (59.1)		132 (57.9)
- Engagement				47 (20.6)
- Technical				46 (20.2)
- Confidentiality concerns				3 (1.3)
- Online format (not specific)		47 (40.9)		
HART in general	123 (46.8)			
- Time too long		43 (35.0)		
- Large group experience		13 (10.6)		
- Disorganized		11 (8.9)		
- Time too short/time in general		8 (6.5)		
- Not relevant		7 (5.7)		
- Too early in training		7 (5.7)		
- “Too Heavy”		7 (5.7)		
- Too personal		7 (5.7)		
- Too much silence		6 (4.9)		
- Small group experience		5 (4.1)		
- Too much religion		5 (4.1)		
- Too focused on grief		4 (3.2)		
Everything valuable			25 (9.5)	
No difficulties			186 (44.9)	
Total coded references	263		414	

Of 435 student responses, 414 references shared challenges with the online format (Q31, Table 4). Just over half of the references cited difficulties and just under half of the references indicated there were no difficulties. Of the references citing difficulties specific to online format, over half indicated difficulties with emotional connection; difficulties with engagement, technical difficulties, and confidentiality concerns were also mentioned.

Within 428 student responses, 440 references shared how the online learning format compared to an in-person learning experience. Half were coded as negative (221/440, 50.2%), and about one-quarter each were coded as balanced (121/440, 27.5%) or positive (98/440, 22.3%).

Exemplary responses to difficulties related to online HART included, “It feels harder to connect with someone over a monitor” and “There is something about feeling someone’s physical presence when you share a story. This could be simply physical proximity to a person, eye contact, a touch of the arm, or a hug. I missed those experiences in the online environment,” “Some Zoom lags made it hard to talk without talking over others,” and “We had a group member with bad internet, and people were more prone to being distracted by things at home, which is normal.”

Exemplary responses related to positive or neutral online HART included, “It would have been nice to meet everyone but I think we were all able to still share deeply over the online format,” “I liked being able to attend from the comfort of my own home,” “Surprisingly, I thought the online nature of the class would be cumbersome or another ‘annoying Zoom’ but I actually enjoyed it thoroughly. Felt very different than the other online events I have for school,” “I don’t have an in-person version to compare it to, but I thought it was still a hugely beneficial experience full of connection,” and “I felt it was ok and if anything, may have been a bit easier to be vulnerable than in person.”

## Discussion

Our three main research questions for this study were the following: (1) What are the experiences of students in an online HART course? (2) What are the challenges for students specific to online HART delivery? and (3) How do students’ learnings compare with the in-person course? Our findings indicate that students who participated in the online HART course experienced professional identity formation, developed a sense of community, and were provided with tools and space for reflection and self-care (Table 3). These experiences, while not exact replicas of the in-person experience, mimic many of the affective skills reported as acquired in the in-person iterations [7, 8, 11, 18, 28, 29]. Evaluation responses indicated that the biggest challenge to the online HART delivery was connection (Table 4), but for many digital natives, online instruction poses few barriers to participation. Finally, our data confirm that similar to the in-person course, HART’s affective-domain online curriculum is effective in supporting students’ professional identity formation [28], developing a sense of community, and helping students to consider tools for reflection and self-care. Our findings go a step further by indicating that online HART is comparable to in-person HART in terms of student growth, satisfaction, and perceived outcomes, as there were no significant differences between responses from online and in-person cohorts (Table 1).

One of the most important implications of our current study is the generalizability of our findings to other courses and situations. While we show that online HART specifically is comparable to the in-person version, instructors of other affective domain courses may choose to explore the transferability of these findings. Professional identity, community, self-care, and peer/patient relationships are no longer acceptable as part of a hidden curriculum; schools are actively pursuing the explicit teaching and reflection of these attributes. As medical and other health professional schools seek to explore the use of online affective-domain curriculum, below we share our successes and challenges.

### Successes

#### Community

Students built a sense of community online, which was invaluable during the pandemic. The many impacts of COVID-19 on medical students around the globe have been illustrated elsewhere [30–35]. As we reflect on what effect the pandemic had on health professional students from the class of 2024, we realize again the value of the community-building inherent in the Healer’s Art curriculum. Even though the course was offered in an online format and despite “zoom fatigue” by early 2021, students signed up for the Healer’s Art to meet their stated need for community. “I have learned how to approach deeper conversations with my classmates and how to develop stronger relationships with my friends, but also how to apply this to patient care and being empathetic with a patient and meeting them where they’re at.” The small group experiences of HART allowed students to engage more deeply and meaningfully with peers and facilitators. Building community is also critical at a time when many medical students feel intense competition among classmates. Instead, HART students experienced a supportive network of peers, “The most valuable thing I learned was that I am not the only one feeling the way I do. Medical school in a pandemic can be pretty isolating. Finding people who share similar goals and values was super valuable for me. I feel a lot less alone now. It also helps me stay committed to patient centered care and remaining a human in this profession because I know others who have that same goal in mind. I have people to lean on in the future and to encourage along the way.” Our findings demonstrate the presence of community in an online setting and we note that HART is highlighted in the US Surgeon General’s Advisory on Building a Thriving Health Workforce as an example of medical trainee education that “helps students stay connected to their core values and humanity and equips them with tools to manage moral injury and stress” [36]. Additionally, the US Surgeon General’s Advisory on the Healing Effects of Social Connection and Community is germane [6].

#### Professional Identity Formation

While most medical school content focuses on basic and clinical science, HART allows students to explore finding meaning in medicine, even in an online setting. Our study shows the ability of an online experience to support students in professional identity formation comparable to in-person HART (Table 1). In the virtual HART, students were able to consider professional identity formation and how to bring their whole self to their profession, as one student mentioned, “There is much more to practicing medicine than physiology, anatomy, and writing notes in the EHR. Connecting with other humans transcends the pure science and allows us to impact others on a personal level.” This online experience has fostered students’ professional identity at both the individual level (based on what they can bring to the table) and at the collective level (the meaning of medicine), suggesting that online professional identity curricula are effective [16]. Enabling students to explore the range of self they bring to the table can reaffirm their commitment to the profession, thus decreasing burnout [37–39]. Effective professional identity work in the curriculum needs opportunities for students to engage in their own identity formation as well as providing a safe and welcoming community for this self-work [15, 40]. The online HART curriculum addresses both through personal introspection and the community aspect of the course. One of the most critical aspects of professional identity formation that students mentioned in their responses was the skill of listening and being more present, as illustrated by this student, “Healer’s Art, more than anything, taught me the value of listening.” The practice of “generous” listening with patients enhances the relationship, encourages meaningful sharing by the patient, and enhances well-being [41]. Even in the virtual setting, HART opens students to the thinking and feeling of being healthcare professionals, an essential element in medical education [15, 41].

#### Self-care Toolbox

At the end of the course, students identified and committed to using self-care tools to build resilience during their careers. For example, one student recalled, “we focus much more on exploring ourselves: how we can connect with our love, passion and emotion, and how we can open ourselves to the beauty, difficulty, sadness, and awe of medicine. In other courses, our small groups talk about how we can be better providers, show up for our patients, and hone our technical skills. But it’s never about bettering ourselves. I’ve realized that’s so important.” This quote, and others, suggests that even in an online setting, the HART curriculum enhances development of self-care practices, tools necessary to combat burnout in healthcare professions [38, 42].

#### Accessibility

Another mentionable success is that many students enjoyed the accessibility and comfort of attending HART from their own homes. Students discuss challenging topics such as grief and loss, which may be even more difficult while sitting in a classroom. One student said, “Being at home definitely makes it feel like a safer space, and a more comfortable environment.” Students mentioned that they felt “braver” from home and could more easily keep in touch with their groups online, further cementing the community aspect of HART online. Students further identified the pluses that came with online HART: “The benefits of the online format (out-of-state participants, no commuting, comfort of home) outweigh any of its negative elements.” While technology tends to be wrought with difficulties [20, 26], the virtual setting of HART allowed students to engage more authentically and more comfortably.

### Challenges

#### Emotional Connection

While many students reported that they experienced no difficulties (*n* = 186) with the online format, the most commonly reported difficulty was emotional connection. This can be a valid concern when interpersonal engagement is a key component to the course. Students said that they had challenges with assessing body language of classmates, which could become a barrier to sharing personal stories and contributing to a discussion. “Obviously, the lack of having a physical presence …dampened the emotional experience a little. However, I feel like we overcame that by simply creating a safe space and encouraging an environment of listening and thinking.” In this case, although students felt less emotionally connected to their peers, the groups were able to overcome that barrier and encourage meaningful participation. Another student said, “There were times I wished I could have hugged my peers so they felt even more connected with me,” indicating that connection existed, but students missed the physical closeness to provide comfort. Some students mentioned the struggle of being unable to assess body language, “I felt that sometimes the silence felt more uncomfortable virtually than it would in person. In person, b/c we can see beyond ‘talking heads’, it’s easier to see visual cues that others are in the process of reflection or thinking.” Even though a large proportion of students cited emotional connection as a challenge, we also show that community and relationships are huge successes in HART online. Despite the challenges, students are getting the value of connection. We believe that the top three ways to overcome this challenge are to encourage a safe space for sharing, to encourage use of video during the class, and to implement warm, personable small group experiences.

#### Engagement

Zoom fatigue was another challenge mentioned. One student said, “The only challenge was that I find it harder to stay engaged for 3 h with a screen vs in person. This had nothing to do with the preceptors, and everything to do with too much screen time.” Many commented on the difficulty with focusing and participating during a long online class.

#### Technical

Although technical difficulties (weak Wi-Fi connections, dropped connections) were mentioned, the number did not rise to the level indicating a significant barrier to the experience.

## Strengths and Limitations

Major strengths of this study are the sample size (*n* = 1224) across 31 institutions and the mixed methods approach. We used the same evaluation instrument at every institution and across multiple years of the study, enabling us to make comparisons across institutions and time. The COVID pandemic forced institutions to adopt online learning, and this is both a strength and limitation; there was no inherent selection bias toward online HART, thus increasing the trustworthiness of our results. We expect that, should students choose online HART, their satisfaction would hold. Future directions might investigate the satisfaction and engagement when students voluntarily select for online or in-person courses.

Regarding limitations, we did not analyze qualitative responses of the in-person cohort, and our argument’s validity may have been stronger. However, we cite published studies that sufficiently describe the in-person experience [7, 8, 12, 18, 28, 29]. Additionally, we recognize that using larger sample sizes within institutions would have allowed examination of differences between online and in-person cohorts within institutions.

## Conclusion

The purpose of this research was to understand the experiences of students engaged in online HART. In all, we found that the outcomes were comparable to the in-person HART. We confirmed that HART provides support for development of professional identity formation in health professions students [28]. It had been unknown if the course would be as effective in an online format. Based on our study, health professions schools that choose to offer HART online can do so with confidence that it will provide students comparable outcomes to in-person learning. In particular, schools with distributed campuses or in large urban areas with challenging commutes can provide the course and anticipate the benefits found with in-person delivery: community, skills in self-care and relationship-building, and overall professional identity formation, required for lifelong learning, well-being, and role satisfaction.

## Supplementary information

Below is the link to the electronic supplementary material.

Supplementary file 1 (DOCX 16.8 KB)

## Data Availability

The data analyzed in this study are not publicly available due to student and instructor confidentiality.

## Declarations

### Data

Schools participating in HART provide collated reports of end-of-course student evaluations to the Remen Institute for the Study of Health and Illness; permission for analysis of this routinely collected data was obtained from individual schools’ HART course directors.

### Disclaimers

The views expressed are those of the authors and do not represent those of the Department of Veterans Affairs or the United States Government.
