# Supplementary material for: Healer’s Art in the Online Era: Successes, Challenges, and Implications
Source: Med Sci Educ. 2025 Jan 13;35(2):977–89. doi: 10.1007/s40670-024-02272-w (PMC12058631; doi:10.1007/s40670-024-02272-w)
Supplement: Supplementary file 1 — (DOCX 16.8 KB) [file 40670_2024_2272_MOESM1_ESM.docx]

**Supplementary File 1.** Schools included in the study.

Baylor College of Medicine

California Northstate University College of Medicine

Creighton University School of Medicine

Des Moines University College of Osteopathic Medicine

Donald and Barbara Zucker School of Medicine at Hofstra/Northwell

Frank H. Netter MD School of Medicine at Quinnipiac University

Northern Arizona University Doctor of Physical Therapy Program

Sidney Kimmel Medical College

Stanford University School of Medicine

Technion American Medical School

Touro University College of Osteopathic Medicine

Tufts University College of Veterinary Medicine

University of California, Los Angeles David Geffen School of Medicine

Uniformed Services University of the Health Sciences

University of Alberta Faculty of Medicine & Dentistry

University of British Columbia - Island Medical Group

University of Buffalo Jacobs School of Medicine

University of Massachusetts Medical School

University of Michigan Medical School

University of Missouri School of Medicine

University of Missouri School of Veterinary Medicine

University of New Mexico School of Medicine

University of Texas - Austin - Dell Medical School

University of Texas Health Sciences Center at San Antonio Long School of Medicine

University of Utah School of Medicine

University of Washington School of Medicine

University of Wisconsin School of Medicine and Public Health

UTHealth McGovern Medical School

Vanderbilt University School of Medicine

Wright State University Boonshoft School of Medicine

Yale University School of Medicine
